# Severe Acute Respiratory Syndrome Coronavirus 2 RNA Detected in Blood Donations

**DOI:** 10.3201/eid2607.200839

**Published:** 2020-07

**Authors:** Le Chang, Lei Zhao, Huafei Gong, Lunan Wang, Lan Wang

**Affiliations:** National Center for Clinical Laboratories, Beijing Hospital, National Center of Gerontology;; Institute of Geriatric Medicine, Chinese Academy of Medical Sciences, Beijing, China (L. Chang, Lunan Wang);; Wuhan Blood Center, Wuhan, China (L. Zhao, Lan Wang);; Shanghai Haoyuan Biotech Co., Ltd, Shanghai, China (H. Gong);; Peking Union Medical College Graduate School, Chinese Academy of Medical Sciences, Beijing (Lunan Wang)

**Keywords:** respiratory infections, severe acute respiratory syndrome coronavirus 2, SARS-CoV-2, SARS, COVID-19, 2019 novel coronavirus disease, zoonoses, viruses, coronavirus, blood donors, China

## Abstract

Because of high rates of 2019 novel coronavirus disease in Wuhan, China, Wuhan Blood Center began screening for severe acute respiratory syndrome coronavirus 2 RNA on January 25, 2020. We screened donations in real-time and retrospectively and found plasma samples positive for viral RNA from 4 asymptomatic donors.

Because of the rapid increase of cases of 2019 novel coronavirus disease (COVID-19; *1*) and detection of severe acute respiratory syndrome coronavirus 2 (SARS-CoV-2) RNA in plasma (*2*,*3*), the safety of China’s blood supply became a major concern (*4*). Most blood centers and blood banks in China began taking measures to ensure blood safety (*5*); on January 25, 2020, we began screening all donations collected at the Wuhan Blood Center. 

We performed real-time reverse transcription PCR (RT-PCR) for SARS-CoV-2 RNA by using MultiScreen Pro RT-PCR assay (SYM-BIO LifeScience, https://www.sym-bio.com.cn). We performed pool testing by mixing plasma from 6–8 samples or individual testing by using 1.6 mL of plasma. We eluted 100 µL of nucleic acid template and added 40 µL of it to the RT-PCR mix. 

By March 4, we had screened 2,430 donations in real-time, including 1,656 platelet and 774 whole blood donations. We identified the first positive donor in our center in a positive pool with a weak amplification of the open reading frame 1ab gene. The donor gave 2 units of platelets on January 28, which were included in the pool. However, the donor’s prior donations collected on December 12 and 26 and January 13 were negative for viral RNA. Hubei Province Center for Disease Control and Prevention performed follow-up tests on plasma on February 2, which showed a weak positive result near the limit of detection; a throat swab specimen collected from the donor on February 10 also was positive, indicating an extremely low viral load in plasma. The donor reported no symptoms and was quarantined in a cabin hospital in Wuhan until 2 consecutive negative throat swab results on February 23 and February 25 (Figure). 

**Figure:**
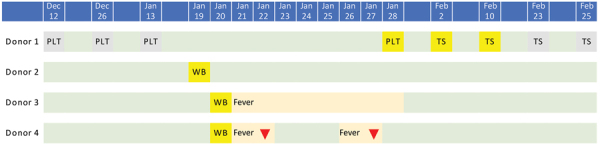
Timeline of donations and symptom onset of 2019 novel coronavirus disease from 4 blood donors, China. Gray indicates a negative result for severe acute respiratory syndrome coronavirus 2 (SARS-CoV-2) RNA; yellow indicates a positive result. Green indicates the donor was asymptomatic or their temperature returned to normal; orange indicates fever; red triangle indicates the donor’s fever subsided after taking self-prescribed antipyretic medications. PLT, platelet; TS, throat swab; WB, whole blood.

We also performed retrospective testing of 4,995 donations collected during December 21, 2019–January 22, 2020, by using retained nucleic acid template after routine pool testing. On February 10, we found a positive result in a nucleic acid template derived from donations collected on January 19. We individually tested samples that were in storage at 2°C–8°C for 23 days because no plasma samples stored at −20°C were available. We identified another positive donor of whole blood. We tested plasma products from his donation twice and noted similar results, which suggests that viral RNA is relatively stable in plasma (Appendix Table). We immediately traced all blood products produced from donor 2’s whole blood, and they had not been used. Telephone follow-ups on February 15 and 25 showed donor 2 remained asymptomatic and quarantined at home.

In telephone follow-ups with donors who gave blood during January and February, we identified 33 donors who developed a fever after donation; all of their donations were removed from circulation. We performed retrospective individual screening on frozen plasma products from 17 donors and tested the retained nucleic acid templates after routine pool testing of the other 16 donors. We found 2 more positive donors who donated whole blood on January 20. Both had weak positive results, and donors reported fever onset on January 21 (Figure; Appendix Table). Donor 3 treated patients infected with SARS-CoV-2 in a Wuhan hospital. His temperature returned to normal 8 days after donation. Donor 4’s temperature also returned to normal 7 days after taking self-prescribed antipyretic medications. 

By March 4, we identified 4 blood donors in Wuhan whose plasma samples tested positive for SARS-CoV-2 RNA (Figure; Appendix Table). Samples from these donors were further tested for specific IgG and IgM against SARS-CoV-2 by ELISA; results were negative, indicating the possibility of infection in the early stage and the need to follow-up with these donors. 

We found SARS-CoV-2 RNA in plasma during routine screening of blood donors, considered a healthy population. We tested the 4 donors multiple times, using different sample sources, including sample tubes, retained nucleic acid templates, or blood products, indicating the accuracy and validity of our results (Appendix Table). One limitation of our study is that we did not have more detailed information on donors 2, 3, and 4. Although we could not confirm virions in blood or whether the virus could be transmitted in blood products, the potential risk should not be neglected. However, detectable RNA might not signify infectivity. Further studies, such as virus culture, should be done to explore the possibility of viremia and follow-up of donors also is essential. 

Of note, the donors all donated in late January, and we did not detect SARS-CoV-2 in plasma samples after then, indicating the strict containment measures taken by the government of China were effective. In China, donors are screened for related symptoms and asked if they feel healthy when they donate blood. Having donors call the blood donation center if they have any symptoms after donating is essential to avoid the risk of donation during the COVID-19 incubation period. Moreover, as more asymptomatic cases occur, screening donors for viral RNA with high-sensitivity assays, as we are doing in Hubei Province, will be critical to ensure blood safety.

**Appendix.** Additional information on severe acute respiratory syndrome coronavirus 2 RNA detected in blood donations.
