# Supplementary material for: Severe Acute Respiratory Syndrome Coronavirus 2 RNA Detected in Blood Donations
Source: Emerg Infect Dis. 2020 Jul;26(7):1631–3. doi: 10.3201/eid2607.200839 (PMC7323524; doi:10.3201/eid2607.200839)
Supplement: Supplementary file 1 — Appendix. Additional information on severe acute respiratory syndrome coronavirus 2 RNA detected in blood donations. [file 20-0839-Techapp-s1.pdf]

Article DOI: https://doi.org/10.3201/eid2607.200839

Severe Acute Respiratory Syndrome Coronavirus 2 RNA Detected in Blood Donations

Appendix

Appendix Table. Information and reverse transcription-PCR results on samples from asymptomatic blood donors tested for severe acute respiratory syndrome coronavirus 2, China*

					Cycle threshold		
Donor	Sex	Age	Donation date	Sample source†	ORF1ab	N	Detection date
1	M	53	2020 Jan 28	A	37.405	36.635	2020 Jan 28
				B	34.346	34.004	2020 Jan 28
				C	34.423	34.577	2020 Feb 7
2	M	37	2020 Jan 19	D	40.219	39.834	2020 Feb 10
				B‡	UD	38.715	2020 Feb 13
				E	38.254	37.655	2020 Feb 14
				E	38.495	37.189	2020 Feb 14
3	M	42	2020 Jan 20	E	UD	40.183	2020 Feb 15
				E	38.268	UD	2020 Feb 16
4	F	21	2020 Jan 20	E	37.607	UD	2020 Feb 7
				E	38.732	37.015	2020 Feb 8
				E	38.644	UD	2020 Feb 8

*The limit of detection of Pro RT-PCR assay (SYM-BIO LifeScience, https://www.sym-bio.com.cn) is 10 copies/mL in 1.6 mL of plasma. Clinical sensitivity and specificity were >99.99% based on donation screening data in Wuhan, China and no cross-reactivity was found with other human coronaviruses, influenza viruses, common human viruses, or transfusion-transmitted pathogens. Cycle threshold of a positive result is ≤42 for 1 region and ≤45 for the other region. Any other situations for any amplification of the 2 regions, such as only 1 region detected or cycle threshold of the 2 regions both were between 42 and 45, the specimen should be retested. N, nucleocapsid region; ORF, open reading frame; UD, undetected.

†A, screening sample tube (pool testing); B, screening sample tube (individual testing); C, platelet product (individual testing); D, retained nucleic acid template after routine pool testing; E, frozen plasma product (individual testing). Pool testing was performed by mixing plasma from 6–8 samples. Individual testing used 1.6 mL of plasma samples. A 40 µL volume of nucleic acid template from 100 µL of nucleic acid eluted was added to the RT-PCR mix.

‡Because of limited sample volume, we diluted the sample 4-fold before testing.
